# Costs in conservation: Common costly mistakes and how to avoid them

**DOI:** 10.1371/journal.pbio.3002676

**Published:** 2024-06-10

**Authors:** Vanessa M. Adams

**Affiliations:** School of Geography, Planning, and Spatial Sciences, University of Tasmania, Hobart Tasmania, Australia; Arizona State University, UNITED STATES

## Abstract

There has been an increasingly prevalent message that data regarding costs must be included in conservation planning activities to make cost-efficient decisions. Despite the growing acceptance that socioeconomic context is critical to conservation success, the approaches to embedded economic and financial considerations into planning have not significantly evolved. Inappropriate cost data is frequently included in decisions, with the potential of compromising biodiversity and social outcomes. For each conservation planning step, this essay details common mistakes made when considering costs, proposing solutions to enable conservation managers to know when and how to include costs. Appropriate use of high-quality cost data obtained at the right scale will improve decision-making and ultimately avoid costly mistakes.

## Abstract 2

More than two decades have passed since the initial push to consider economic costs in conservation. This Essay investigates why more progress has not been made and highlights common mistakes made when selecting, estimating, and including costs in conservation decisions.

## Introduction

With increasing demands on natural resources, such as clean water, food, and fuel, it is becoming critical that we manage ecosystems to sustain the supply of these goods and services [1]. To achieve this, a primary focus of conservation has been designing and implementing area-based conservation measures. Countries have committed to such actions through international mandates such as the Convention on Biological Diversity [2,3]. Delivering on these mandates will require an understanding of where conservation is needed and what must be done to achieve it, both within the protected and conserved estate and beyond. Furthermore, social and economic considerations are crucial to ensuring that conservation measures deliver both biodiversity and social outcomes [4].

Over the past 35 years, the technical ability to design spatially explicit conservation interventions has advanced significantly [5]. The relative importance of considering data about costs in conservation planning became a particular focus of academic publications following the seminal work by Ando and colleagues [6], which demonstrated that cost-effective selection strategies achieve the same species coverage at lower costs compared to those that ignore heterogenous costs [6]. This work and others emphasized that including economic costs into conservation planning had the potential to improve outcomes through cost-efficient design of conservation actions [7–9] and estimating the true financial needs of conservation programs for appropriate resourcing [10–13]. The benefits of including costs in conservation planning were highlighted in an overview of the field [14], which also summarized best practice economic methods for modeling spatial explicit conservation costs and called for expanded adoption of such econometric frameworks to improve the type of cost data considered in conservation (see Box 1 for a definition of the types of conservation costs). In other words, including costs in decision-making should allow conservation to achieve more with less. However, a key point is that most observed “cost-efficiencies” observed are attributed to the fact that conservation costs are much more spatially variable than biodiversity features [6,7,15–17], and the more variable data will drive the spatial selection of priorities [7,14,18].

**Box 1. Glossary**

Conservation costs can be separated into 5 components: acquisition, management, opportunity, transaction, and damage [14]. This essay focuses on the financial costs of conservation most frequently estimated and considered in conservation planning: acquisition, management, and opportunity costs. Each is defined below noting common estimation methods or surrogate measures used in the conservation literature.

**Acquisition costs** are the costs of acquiring a bundle of rights from a rights holder. Most commonly these are related to parcels of land and are therefore relevant to terrestrial conservation planning; purchase of rights can also occur in marine environments as well such as via the transfer of licenses for fishing or closures to traditionally managed marine areas. In the traditional definition of acquisition costs—relating to the purchase of land parcels—acquisition costs are best estimated using historic sales records and hedonic modeling which accounts for factors influence property prices [19–21]. However, sales records can often be challenging to access due to the commercial in confidence nature of such data. Land value is more commonly used as a surrogate measure for acquisition costs and either land valuation records that have complete spatial coverage are used, based on government records, or hedonic modeling is used to estimate missing data [22]. Where sales and land value records are absent or unreliable—for example, in countries that do not have land tax systems—another common surrogate used is estimated opportunity costs.

**Opportunity costs** are the costs of forgone opportunities associated with changed management or use of a place (e.g., protecting or conserving and thus reducing future extractive uses). Here, we focus on opportunity costs from lost production (hereafter simply referred to as “opportunity costs”); these can be separated by different types of extractive uses to stakeholder groups and thus the relative distribution of costs across groups [23]. In terrestrial planning, opportunity costs are commonly estimated using land use modeling and current commodity prices [24,25], while in the marine environment, methods to estimate have included fishing models and market prices specific to particular target catches [26] and stakeholder mapping approaches [27,28].

**Management costs** are those associated with the ongoing maintenance of a conservation program and can be broken down into fixed costs, which are independent of the amount of conservation effort, and variable costs, which are proportional to the type or amount of conservation management [29]. Management costs can be a significant portion of total conservation costs and are therefore often considered in developing conservation plans. Complete cost accounting, capturing context of the management action as well as the specific activities, frequency, and timing, provides the most reliable measure of calculating the financial costs of management [30]. The benefit of such accounting is that the data can also be used by others to estimate the costs associated with management in similar contexts. Despite the benefits of such reporting and data sharing, the practice remains rare. Agencies more frequently report management costs in aggregate, or at a per unit level, making transferability of such data limited. Where site specific estimates of management costs are absent a common surrogate is to assume management costs will be a ratio of acquisition costs—an assumption that has been shown to not hold [20,31] but unfortunately remains common in practice.

A precautionary lesson is that, if cost data truly drive optimal conservation allocations, we need a more complete understanding of the distribution of costs [20,31–35]. In simple terms—the hoped for gains in conservation are reliant on integrating cost data that accurately capture fine grain patterns. This is an essential observation that supports the need to include cost considerations in conservation, but also underscores the need for high-resolution, quality data. Conversely, including the wrong cost data can have negative consequences for conservation decisions [20,36,37].

**Fig 1:**
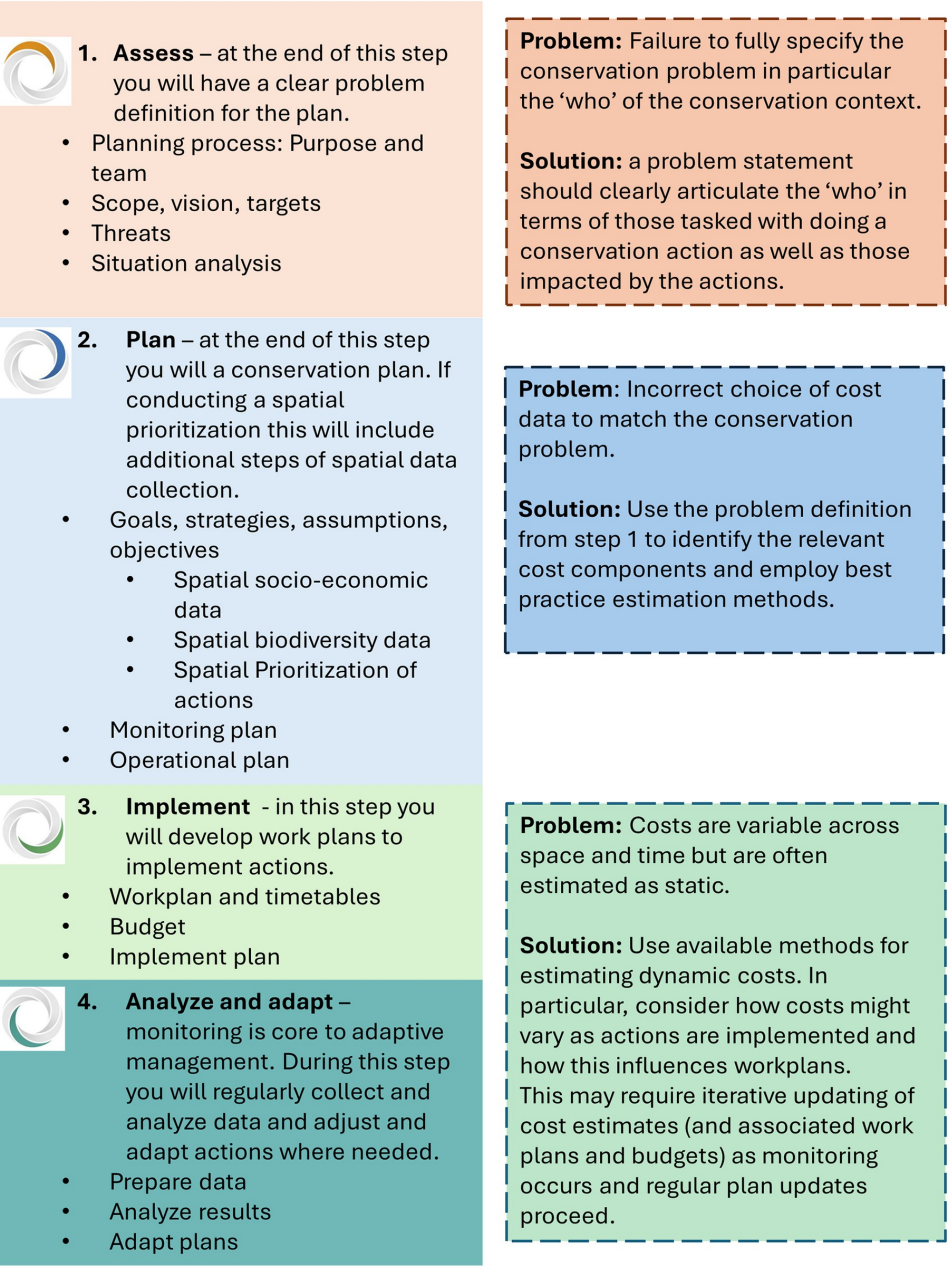
Conservation planning steps and associated problems. The conservation planning steps on the left are adapted from the Conservation Standards (solid boxes [38]) and the associated problems with cost considerations and proposed solutions are on the right (dashed boxes). Each step is defined with subsidiary components.

More than 2 decades have passed since the initial push in the academic literature to consider economic costs in conservation. The importance of data quality, and the consequences of poor cost estimation, have been further highlighted in subsequent reviews [20,37]. However, this academic debate has not translated into changed practice. The common mistakes identified by Naidoo and colleagues [14], and further discussed by Armsworth [20], are revisited here and embedded within conservation planning steps (Fig 1—Conservation Standards [38]). For each planning step, socioeconomic considerations and mistakes commonly associated with them are reviewed and solutions proposed (Fig 1). The solutions are drawn from available methods and tools. The essay aims to demonstrate that the solutions discussed are practical and that conservation decisions will benefit from investing the required resources into defining, estimating, and integrating cost data (with a particular emphasis on the financial costs of conservation, Box 1).

## Step 1: Assess—Identifying the “who” and “what”

The first step of conservation planning is to assess the context; the key output of this step is a well-defined problem statement (Fig 1, Step 1). The essential elements of a problem definition are: the conservation objectives; threats to these objectives; actions based upon a system model that links actions to threat (reduction) and ultimate contribution to meeting the conservation objectives; and resource constraints, which can include the availability of stakeholders or their willingness to participate in a program, funding, costs of actions, as well as broader social values or political aspects of any decisions [39–41]. These can be summarized into the “who” (stakeholders or decision-makers), “what” (actions), and “why” (objectives and threats) of the conservation problem; each component can be linked via a theory of change to clearly articulate the logic for the plan [38,42].

The common mistake at this step is to not fully specify the problem, taking into account the context of implementation and a broader set of stakeholders involved in the conservation plan [39,43]. The solution is simple to express: expand the problem definition to consider more broadly the set of actors impacted by a program and consider alternatives to the status quo of who should lead a plan or program. Identifying the “who” within a problem definition is likely to be iterative, with a first step in the conservation standards being to identify the initial planning team, core decision-makers, and stakeholders empowered within the planning. A later component of Step 1 is to conduct a stakeholder analysis as part of a situation analysis or theory of change. A clear statement of each actor’s action, and associated costs and benefits, then flows logically. For example, in Box 2 alternative problem definitions are posed between conservation organization, government-led, or locally led plans, demonstrating the differences in costs chosen for planning and ultimately the spatial configuration of conservation areas and associated financial costs.

**Box 2. That is not fair. Inequitable distribution of costs when the “who” is not well defined**

A common approach to defining the conservation problem and subsequent choice of cost measures is to focus on the direct costs to the implementing organization, with a desire to minimize these. However, this neglects a more nuanced view of “who” is involved in conservation, and how the impacts or costs to these stakeholders should be considered. This would then influence the choice of cost metric and spatial prioritization of areas.

Consider for example the expansion of the Mbaracayu Forest Biosphere Reserve in Paraguay, where there are several stakeholder groups directly impacted: the implementing conservation organization, local smallholders, local ranching businesses, and multinational soybean producers. Opportunity costs were estimated for each [24] and incorporated into spatial prioritization to consider the spatial design of expanding reserves [23]. Depending on the problem definition and choice of “who” the cost estimates will be different. This then influences the spatial configuration of reserves and associated costs. To demonstrate this, consider 3 scenarios and the relative opportunity costs to stakeholders (see [23] for original analyses and full figures).

**Fig 2:**
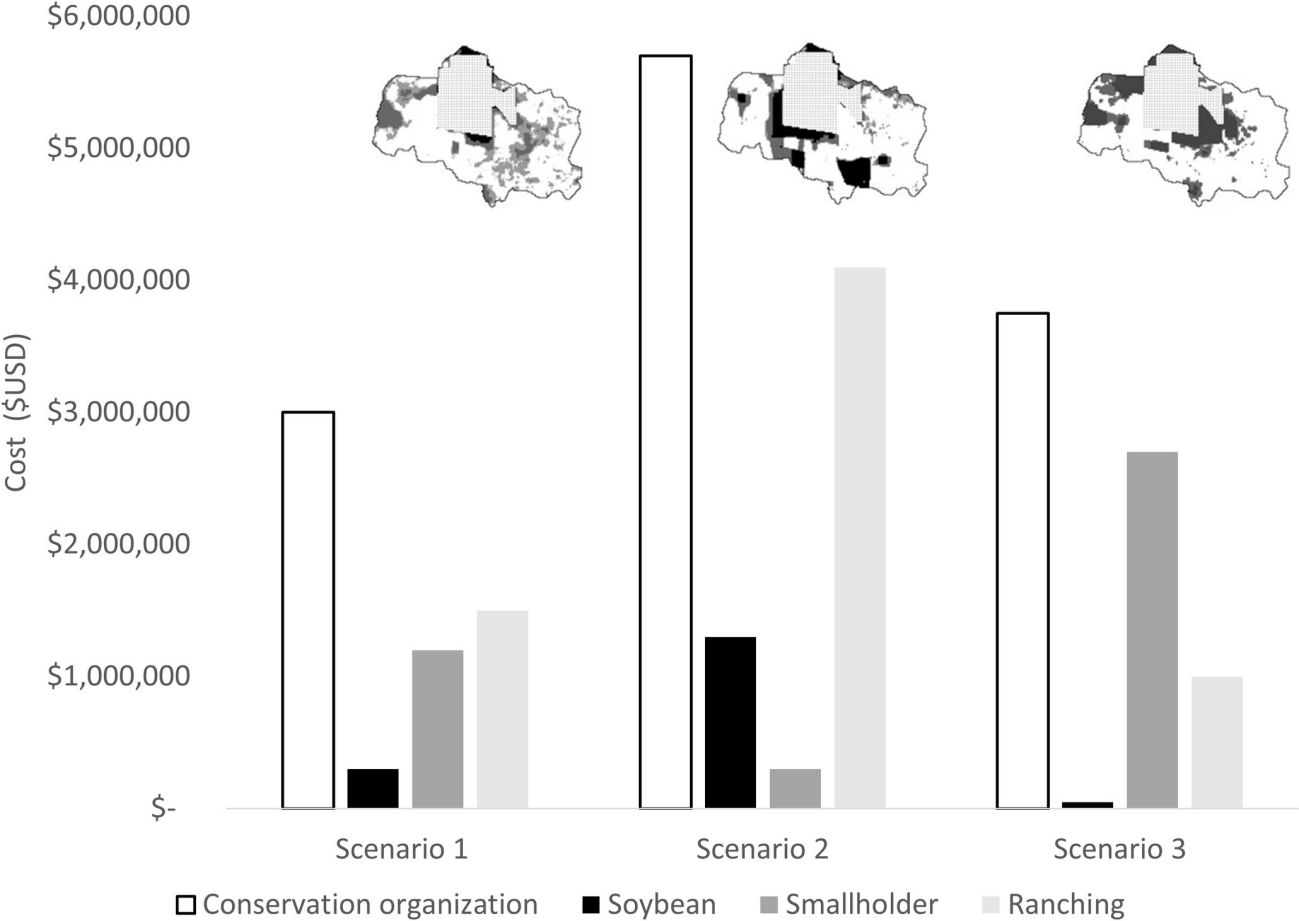
Three planning scenarios and distribution of associated opportunity costs across stakeholders. The data and results are adapted from [23]. For each scenario, a map of prioritized areas for reserve expansion is included above the cost distribution. Scenario 1 minimizes costs to the conservation organization. Scenario 2 minimizes costs to smallholders. Scenario 3 minimizes costs to soybean production.

Scenario 1 reflects the common approach to problem definition which seeks to minimize the costs to the conservation organization. In this scenario, the appropriate cost metric to use is total opportunity costs as an estimate of the cost of land acquisition (Scenario 1, Fig 2). While this results in the lowest acquisition costs to the organization the costs ($3 million), the distribution of costs is uneven with costs largely borne by subsistence smallholders ($1.2 million) and local ranching ($1.5 million) (Fig 2).

If, instead, the problem definition explicitly seeks to avoid costs to those who would be most impacted (smallholder opportunity costs as the key cost consideration, Scenario 2; Fig 2) the financial cost of acquisitions to the organization is nearly double ($5.7 million), and the costs are substantially larger to soybean production ($1.3 million). This is a larger cost borne by some stakeholders but is perhaps fairer as those more able to bear the costs have a larger share. This also ensures that those most critical to on ground implementation (in this case, local community such as smallholders) are less impacted ($0.3 million).

Lastly, in Scenario 3 if the conservation organization seeks to minimize disruptions to supply chain productions or if a government seeks to minimize conflicts with multinational organizations, then the problem definition would be to minimize costs to soybean production and the cost component included would be opportunity costs for soybean. In this scenario, the costs are negligible for soybean production ($0.05 million) and instead displaced on local community (smallholders and ranching, $3.7 million collectively, Scenario 3; Fig 2).

This example emphasizes that failing to consider the distribution of costs across stakeholders may result in inequitable distribution of cost burdens. In addition to the costs varying significantly across stakeholders, the spatial prioritizations also vary. This is due to the spatial patterns of land use and opportunity costs across stakeholders (Fig 2).

Being clear about the pathways to impact for conservation success, and the relative costs and benefits borne by stakeholders, are key considerations for a clear problem definition (Step 1 Assess–Fig 1). This enables appropriate choice of cost measures and subsequent considerations of how costs are distributed across stakeholders. Incomplete or inappropriate problem definition comes at the risk of designing unjust programs which cause social harm to particular segments of the population.

Success in Step 1 underpins the next step—selecting the correct data and considering the appropriate distribution of costs and benefits across actors (Box 2). The challenge in this solution is not a methodological one—the methods for stakeholder analysis are widely used in conservation practice [44,45]. Rather, the barriers may be institutional when considering how to empower or integrate impacted stakeholder groups into planning and program delivery.

## Step 2: Planning with the right data for the conservation problem

The next step in the planning process is to conduct the planning, including a prioritization of actions (spatial or aspatial), based on the problem definition (Fig 1, Step 2). Step 1, when done well, results in a clear articulation of “who” is tasked with doing a conservation action, as well as “who” the impacted stakeholders are. This then guides the choice of costs to include in Step 2, as well as a consideration of how costs are distributed across stakeholders, and potential equity concerns [23,46]. Neglecting these considerations can result in spatial prioritization that negatively impacts stakeholder groups resulting in inequitable outcomes or social harms [23,26,27] (Box 2).

In assembling appropriate conservation and economic data for the plan, a key consideration is that the relative variability of cost and benefit data will influence spatial priorities. Therefore, modeled data should reflect the true variability and distribution of costs [20,31–35]. Key mistakes in the planning step belong to 2 categories: wrong choice of cost components, which is often based on the assumption that cost components are correlated and act as surrogates; and inappropriate use of coarse-scale data, resulting in estimated costs that do not capture the true cost variability and distribution.

### Wrong choice of cost components

A pervasive issue plaguing this step is which cost components to consider and include. The cost components most frequently included are opportunity and acquisition costs, often because the data are more readily available to planners. Data on other cost components are underreported, or tracked in ways that hinder usefulness, e.g., reported in aggregate across large spatial scales or across multiple distinct programs, thus masking actual activities, heterogeneity in drivers, and distributions across stakeholders. This is particularly problematic for management costs, which can occur in perpetuity and thus dwarf acquisition costs.

Due to this bias in the type of data reported and easily accessed, studies have relied on the (often realistic) assumption that acquisition and management costs are positively correlated, or that management costs are uniform across space. Thus, including heterogeneous acquisition costs would be sufficient to capture the total cost structure. This assumption has been debunked and its impacts on spatial priorities and program costs demonstrated [20,47].

### Inappropriate spatial grain of data

Once the cost components have been selected, the next challenge is to ensure that the data have relevant spatial resolution for the analyses. It is common to rely on readily available data that are far too coarse to be useful in fine-grain analyses, such as using regional or national scale data when prioritizing at local scale for, e.g., property or sub-property level conservation prioritizations [20,36]. For example, prioritizations may include land valuation and agricultural rent data reported at county level averages, while decisions are made at individual (or sub) parcel level, or management costs estimated at state or country level [11,48] for subnational scale decisions.

Including averaged data in decision-making smooths the actual heterogeneity of the costs and substantively alters the overall data structure, i.e., the relationships between costs, benefits, and threats, all of which render the data inappropriate for decision-making [20]. Using such coarse-grain cost estimates alongside fine-grain environmental data has been demonstrated to influence spatial choices, including choosing more expensive configurations than expected, or even missing conservation targets due to avoidance of areas that were misattributed as “too expensive” [20,35,47,49,50].

### Estimation techniques and ways forward

Due to the issues of relative resolution of cost and biodiversity data, not all cost data are equal, and the inclusion of any available data on costs is not necessarily better than omitting data. The quality of data matters. If the cost data available is ultimately unlikely to mirror the heterogeneity of the true costs of conservation, including this data can do more harm than good. The consequences of budgeting with poor data are exemplified in Box 3, demonstrating that these can result in misestimating costs by (at least) an order of magnitude.

**Box 3. How much will it cost?! Budget blowouts as a consequence of hopeful assumptions and incomplete data**

Mis-assigning the types of cost to consider, or assumptions underlying estimates, will result in changes in spatial targeting of priorities with associated large uncertainties in budget estimates [19,51].

For example, in 2008 the Queensland Government announced a policy of expanding their reserve estate to 20 million hectares by 2020. The Government’s cost estimate was $120 million [52]. This estimate was focused on capital acquisitions not ongoing maintenance, thus immediately ignoring core cost components associated with the conservation program. The estimate also relied on wishful thinking with respect to pathways into the reserve estate and the associated acquisition costs to the Government. Adams and colleagues [19] demonstrate that expected costs exceed this and are highly dependent on key assumptions.

The Queensland Government’s pledge included a commitment to purchase 4 million hectares in new reserves. Alongside acquisitions, the Government also signaled an intention to convert state land (either unallocated stated land (USL) or state land with extractive leases to be resolved) to reserves [52]. The vast remainder of the expansion was expected to occur on leasehold land, through a mix of voluntary or compulsory covenants, and freehold land through voluntary Nature Refuge agreements [53]. Estimating the full costs of meeting the target of 20 million ha by 2020 requires understanding the spatial distribution of conservation values and likely reserve designs, which then specifies the acquisition pathway and associated costs (including acquisition, management, and transaction costs). The budget estimates using this approach found that, even under the Government’s hopeful assumptions, a minimum budget requirement is $250 million—twice the original estimate. Depending on a full range of possible assumptions and pathways, the estimated budget ranged from $214 million to $2.9 billion [19].

Key drivers of possible budget increases are: (1) the extent to which subdivision of properties is feasible (such that only desirable conservation values could be purchased rather than whole properties); (2) pathways into the reserve estate; and (3) the conservation targets set. Costs respond nonlinearly to changes in these assumptions. Accurate cost estimations therefore rely on the use of modeling that considers the spatial distribution of conservation features, tenure, program costs (specific to tenure), and property boundaries [19].

Solutions to these persistent traps start with improving the availability of fine-grain data across cost types. Given the relative importance of management costs, priority should be given to widespread adoption of transparent reporting using a standardized framework which details context (including temporal and spatial dimensions) and cost components at the activity and site. The lack of fine-grain reporting hinders progress in understanding the patterns of management costs required to support the programs that NGOs and Governments alike seek to deliver. Although organizations collect financial expenditure data, and often share it for reporting purposes, expenditure tracking is often at aggregated scales that render the data challenging to work with for the purposes of modeling. Instead, time-intensive work to disaggregate these figures to activities or management approaches is required [30]. Adopting recommended reporting approaches to standardize how costs are tracked and shared would reduce major barriers in improving the granularity of cost models [29,54].

If appropriate data (both in activity type and spatial grain) are not available, modeling methods could produce data more likely to reflect the true spatiotemporal variability in underlying data structures [20]. There are many well-documented and familiar ways to elicit and estimate costs, such as stated preference methods (e.g., contingent valuation, choice modeling), hedonic modeling, simulation methods, and mechanistic modeling [14,20,21,55,56].

## Step 3: Implementation over time and space

Step 3 takes the plan produced in Step 2 and develops the required operational work plans to begin implementing and monitoring. Therefore, cost estimates used to develop the plan and budget for it within Step 2 will be the basis for Step 3. Implementation of plans is frequently multiyear and incremental in nature, and thus the spatiotemporal variability of costs and how they respond dynamically over time [55] is essential in moving from plan to implementation.

While the spatiotemporal dynamics of costs have been observed, little guidance has been given as to whether these dynamics can be accounted for during the planning phase of conservation. Additionally, these dynamics will affect the scheduling of conservation actions through time, yet methods for scheduling conservation actions have not accounted for these dynamisms [15,57]. There has been some treatment of how acquisition costs respond to conservation interventions through land market feedbacks [55,58], and of how opportunity costs vary in transitioning land and seascapes [24,26]; however, considerations of dynamic management costs have been relatively neglected.

Management costs are likely to display economies or diseconomies of scale [31,59,60] as well as temporal variability [29,61]. However, as noted above, it is common to ignore both spatial variability and temporal variability of management costs (i.e., how costs vary from startup to long-term maintenance). Ignoring how costs respond to changing contexts over time can easily cause misestimation of management costs; the repercussions include negative outcomes for operational planning and resourcing of management teams.

The solutions here build upon those in Step 2. If costs are reported transparently using standardized accounting methods [30] that specify temporal period as well as spatial context, then improved estimation and modeling methods can be applied. For example, mechanistic models could then be parameterized to estimate the likely costs given the spatial and temporal extent of programs, as well as the optimal allocation of management action type (e.g., aerial or on ground weed treatments, Box 4) [62]. These considerations are familiar to on-ground managers tasked with operational planning, but perhaps less so for those in decision support or conservation prioritization roles that are focused on strategic planning. An explicit connection between these roles is needed to ensure the divide from plan to implementation is not widened by poor consideration of cost structures and the on-ground teams they represent [39].

**Box 4. What are we doing, where, and when? Planning and costing for implementation**

On-ground management actions are highly context dependent. Consider managing an environmental weed capable of establishing large monoculture (>1,000s ha). Eradication within a sparse infestation (<1% coverage) might require on-foot search with limited spray or hand-pulling once individual plants are found [63]. In contrast, controlling a large and dense infestation (>50% coverage) might be more effective with aerial spray, for which costs are largely associated with the hire of highly skilled helicopter pilots [64]. Estimating accurate costs thus requires understanding the context of management. Even within a single technique, such as aerial spraying, there may be substantial differences in the per hectare costs depending on spatial extent of management (i.e., potential for economies or diseconomies of scale) [65].

**Fig 3:**
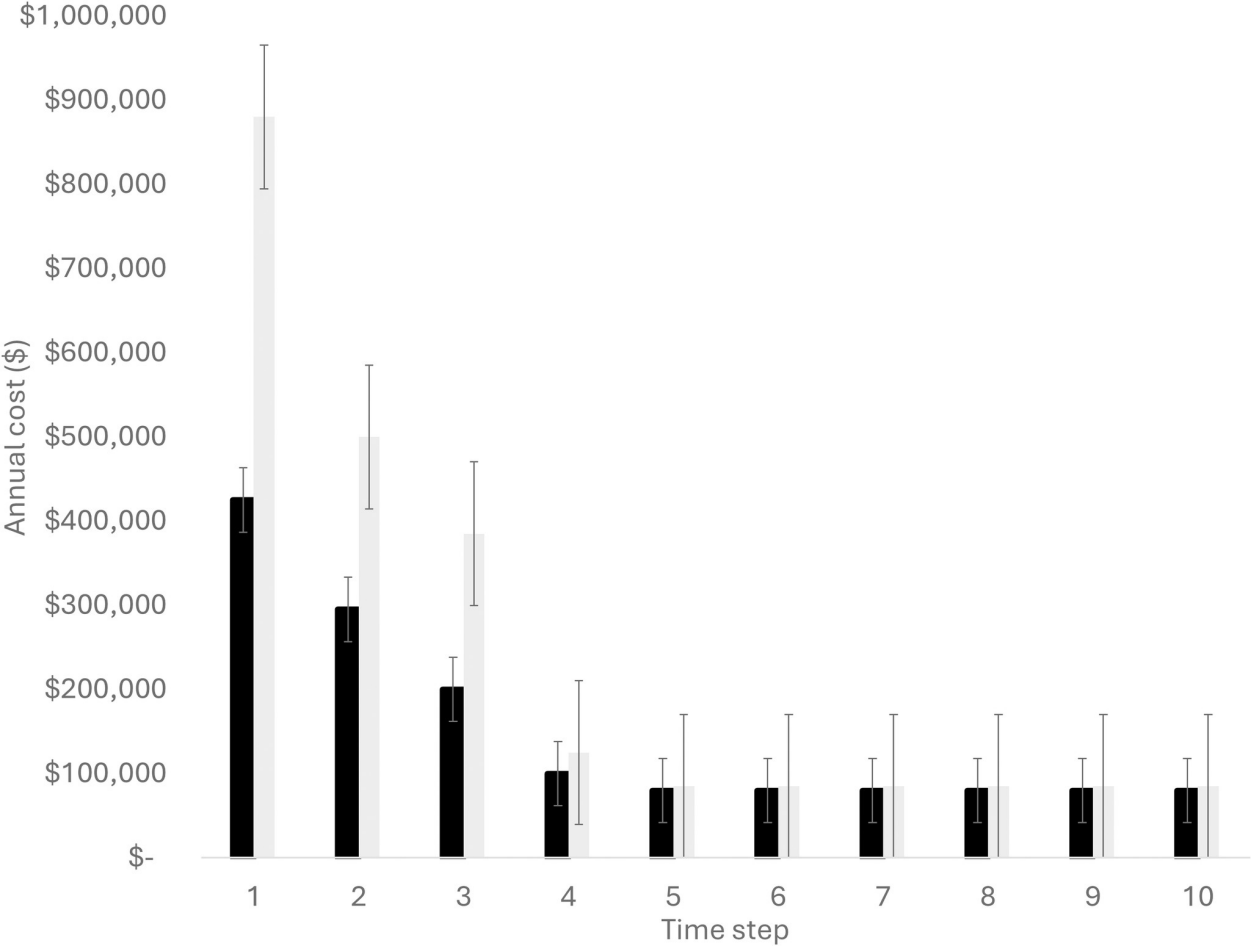
Regional annual weed management costs over time for 2 possible management scenarios. Figure adapted from Adams and colleagues [62] as an example of cost estimates using simulation methods and embedded in-scenario planning and evaluation.

The year of implementation also matters—most management programs require large up-front investments in activities, followed by ongoing maintenance (Fig 3) [66]. Mechanistic models that capture the on-ground activities can be used in spatial targeting or scenario testing, along with building realistic cost estimates [65,67]. Switch points in decisions between deploying different methods (e.g., aerial spray versus on-ground treatment) can also be identified as model outputs, and relative to contextual attributes such as weed infestation size, density, and accessibility.

An agent-based weed spread and management model was developed to test spatially explicit management scenarios [68,69] and has been subsequently used to support management planning in national parks [62,64]. The required data to build the model included: on-ground research spray trials (to test effectiveness and cost the model inputs) [63]; cost quotes and methods of management from a contractor (supplied to support research) [70]; and further expert input from managers on how to approach on-ground management, depending on size and density of infestation. Although collecting these various data sets and validating models require time investment, the necessary data are accessible and can be reliably elicited. A key feature of the model is that it provides both labor hour and financial cost estimates for management scenarios, which supports aspects of work planning in addition to financial budgeting (Fig 1, Step 3—Implement).

Using this approach, the estimated annual total management costs for a regional weed management strategy are likely to vary each year and be dependent on year. There are large labor and financial commitments during the first years of large-scale eradication programs, followed by very low levels of labor time and financial costs in maintanenace years (Fig 3). Using an agent-based modeling approach to design and test management scenarios for overall feasibility and benefits, the cost estimates range from $400,000 to $900,000 a year in the first years of a large-scale eradication campaign to $80,000 a year in maintenance years (primarily costs accrued in monitoring control boundaries; Fig 3).

Modeled annual management costs for different scenarios can provide one point of reference for management planning, alongside the relative benefits of each (e.g., avoided loss of areas to weed infestation, protection of natural values, reducing risk to human life or assets) [64]. Coupling spatial management maps alongside annual cost estimates and labor hours can support managers to move from planning to implementation and ongoing evaluation.

## Ways forward

The above discussion has emphasized the consequences to conservation programs of mis-assigning and estimating costs, with a particular emphasis on the potential to erroneously shift conservation priorities in response to incorrect cost data, or to wildly misestimate program costs. Addressing the quality of economic cost data is important but not a panacea. In addition to the pragmatic imperatives of bringing conservation programs in at a minimum cost, there are broader philosophical reasons for economic costs to be central in decision-making. By including economic considerations in conservation planning, decision-makers must make explicit the multiple (often competing) goals for a place, and in doing so make any trade-offs transparent [32]. It is critical to keep in mind the purpose of cost data and its role in planning: to ensure conservation programs are properly resourced and supported. Where data are judged to be of insufficient quality, for reasons discussed above, an alternative is to use plan processes to reveal costs and benefits.

### Processes alongside data

In many cases, rights holders will know more than conservation organizations about the costs of managing land to deliver environmental services and about opportunity costs to production. In these cases where there is information asymmetry, measuring the true costs of programs a priori may be impractical. A solution is to omit estimated cost data from the planning step, and instead carefully design processes for eliciting costs and benefits of actions. Such processes still benefit from a baseline map of conservation features (e.g., species, ecosystems, ecosystem services) to understand what the values, threats, and required actions are. Depending on the planning problem and the stakeholder roles in planning, this map can then be embedded within processes such as reverse auctions, collective bargaining, or participatory decision-making (e.g., community-driven plans) [43,71–73]. The type of process chosen should be matched to the problem definition and stakeholders just as cost data should be. Examples of context and processes are given here for allocating private land conservation stewardship contracts, public forest resource use, and community-based management.

In the case of private delivery of environmental goods, such as on private land, auctions are a common (but diverse in form) method to elicit costs [73]. Auctions, either pilots or ongoing programs, can reveal appropriate pricing of environmental services [74–76] and other social dimensions of participants [71,77]. It is worth noting that information on environmental benefits, as well as other signals of cost (e.g., land valuation, auction reserves, agricultural rents, and surrogate measures of benefits and/or costs), are still commonly used by those running the auction to evaluate the bids received alongside the elicited costs (for summaries of program inputs, see in particular [71,75]).

In the case of public resource use and management decisions, such as public forest reserves, information asymmetries can also exist across user groups. In this case, given that the stakeholders have shared interests in a public good, collective bargaining can be used to reveal stakeholder preferences and opportunity costs. Such negotiations or collective bargaining require careful design to ensure power structures can be navigated to deliver equitable outcomes [78]. Data still plays a central role as a means of guiding the negotiations; for example, benchmarking known environmental values and social costs attached to particular parcels, and tracking the extent to which conservation objectives are met as collective agreement is reached (e.g., to protect a parcel or leave in forestry leases or sell on) [79]. The NSW Regional Forestry Agreement is a particularly well-documented example of the use of decision support tools and data in a bargaining setting [43,79].

Lastly, there are cases where the true costs of conservation are challenging to estimate because land and sea uses are in transition, or community-based management structures make individual and collective costs hard to disentangle [80]. Participatory, community-driven planning is appropriate in these cases [81]. Data is an important input and used similarly to collective bargaining; for example, maps of environmental values or other identified community values can be used to facilitate community-based decision-making [82,83]. In Kubulau Fiji, a community-based participatory planning process for marine protected areas was chosen over desktop prioritization methods using modeled data. This is an example of where costs had been elicited (including catch per unit effort, profit, and opportunity costs [26]) but were ultimately not fit for purpose and instead a community-led process was designed. The existing data were used as supporting measures for the community-driven process, which allowed for a broader set of values to be considered.

The planning processes highlighted here are indicative and represent a range that can be tailored to different contexts. For further discussion around the choices in participatory planning processes and stakeholder engagement commitments, see Adams and colleagues [43]. A feature of the case studies discussed here is that data on both conservation values and costs play a supporting role, but the final program design emerges from the planning processes.

## Conclusions

There has been an increasingly prevalent message that cost data must be included in conservation planning activities to make cost-efficient decisions [14,17]. However, as argued here, while socioeconomic considerations are a critical component of conservation decisions, data must be appropriate and fit for purpose to be included. Including the wrong data, either in scope, quality, or resolution, can have detrimental effects on conservation programs. While the intractability of some of these issues is troubling—given that they were first highlighted in the literature more than 2 decades ago—the solutions are within the grasp of conservation decision-makers. Data has never been more available and methods for estimating and modeling costs have progressed substantially. Embracing standardized cost-accounting methods for data reporting will further improve the quality of data available, allowing for the estimation of costs controlling for contextual factors. However, improving cost data quality is not sufficient on its own [29,54]. Many of the remaining issues are ones of practice, mainly making socioeconomic considerations equal in weight to environmental ones from problem definition through to program design and evaluation (Fig 1). There are many tools in the planner’s toolbox well suited to overcoming these hurdles that will ensure costly mistakes are avoided, and ultimately conservation decisions are improved.

## References

[1] IPBES. Global assessment report on biodiversity and ecosystem services of the Intergovernmental Science- Policy Platform on Biodiversity and Ecosystem Services. Bonn, Germany: IPBES Secretariat; 2019.

[2] CBD. Kunming-Montreal post-2020 Global Biodiversity Framework. CBD/COP/DEC/15/4. Montreal: Convention on Biological Diversity; 2022.

[3] Secretariat CBD. Global Biodiversity Outlook 5 Montréal. 2020.

[4] Sandbrook C, Albury-Smith S, Allan JR, Bhola N, Bingham HC, Brockington D, et al. Social considerations are crucial to success in implementing the 30×30 global conservation target. Nat Ecol Evol. 2023;7(6):784–785. doi: 10.1038/s41559-023-02048-2 37046146

[5] Margules CR, Pressey RL. Systematic conservation planning. Nature. 2000;405:243–253. ISI:000087080100062. doi: 10.1038/35012251 10821285

[6] Ando A, Camm J, Polasky S, Solow A. Species distributions, land values, and efficient conservation. Science. 1998;279(5359):2126–2128. WOS:000072775600053. doi: 10.1126/science.279.5359.2126 9516117

[7] Ferraro PJ. Assigning priority to environmental policy interventions in a heterogeneous world. J Policy Anal Manage. 2003;22(1):27–43. doi: 10.1002/pam.10094 | ISSN 0276-8739 WOS:000179671600002.

[8] Ferraro PJ. Conservation contracting in heterogeneous landscapes: An application to watershed protection with threshold constraints. Agric Resour Econ Rev. 2003;32:53–64.

[9] Ferraro PJ. Targeting conservation investments in heterogeneous landscapes: A distance-function approach and application to watershed management. Am J Agric Econ. 2004;86(4):905–918. WOS:000224341000003.

[10] Bruner AG, Gullison RE, Balmford A. Financial costs and shortfalls of managing and expanding protected-area systems in developing countries. Bioscience. 2004;54(12):1119–1126. WOS:000225937900010.

[11] Balmford A, Gaston KJ, Blyth S, James A, Kapos V. Global variation in terrestrial conservation costs, conservation benefits, and unmet conservation needs. Proc Natl Acad Sci U S A. 2003;100(3):1046–1050. doi: 10.1073/pnas.0236945100 WOS:000180838100051. 12552123 PMC298723

[12] James A, Gaston KJ, Balmford A. Can we afford to conserve biodiversity? Bioscience. 2001;51(1):43–52. doi: 10.1641/0006-3568(2001)051[0043:CWATCB]2.0.CO;2

[13] James AN, Green MJB, Paine JR. Global review of protected area budgets and staff. Cambridge; 1999.

[14] Naidoo R, Balmford A, Ferraro PJ, Polasky S, Ricketts TH, Rouget M. Integrating economic costs into conservation planning. Trends Ecol Evol. 2006;21(12):681–687. Epub 16 October 2006. doi: 10.1016/j.tree.2006.10.003 17050033

[15] Bode M, Wilson KA, Brooks TM, Turner WR, Mittermeier RA, McBride MF, et al. Cost-effective global conservation spending is robust to taxonomic group. Proc Natl Acad Sci U S A. 2008;105(17):6498–6501. WOS:000255534100051. doi: 10.1073/pnas.0710705105 18413614 PMC2359771

[16] Moore J, Balmford A, Allnutt T, Burgess N. Integrating costs into conservation planning across Africa. Biol Conserv. 2004;117(3):343–350. doi: 10.1016/j.biocon.2003.12.013 | ISSN 0006-3207 WOS:000220702900012.

[17] Polasky S. Why conservation planning needs socioeconomic data. Proc Natl Acad Sci U S A. 2008;105(18):6505–6506. doi: 10.1073/pnas.0802815105 WOS:000255841600001. 18448673 PMC2373354

[18] Babcock BA, Lakshminarayan PG, Wu J, Zilberman D. Targeting tools for the purchase of environmental amenities. Land Econ. 1997;73(3):325–339.

[19] Adams VM, Segan DB, Pressey RL. How much does it cost to expand a protected area system? Some critical determining factors and ranges of costs for Queensland. PLoS ONE. 2011;6(9):e25447. doi: 10.1371/journal.pone.0025447 21980459 PMC3182235

[20] Armsworth PR. Inclusion of costs in conservation planning depends on limited datasets and hopeful assumptions. Ann N Y Acad Sci. 2014;1322:61–76. Epub 2014/06/13. doi: 10.1111/nyas.12455 . 24919962

[21] Adamowicz WL. What’s it worth? An examination of historical trends and future directions in environmental valuation*. Aust J Agric Resour Econ. 2004;48(3):419–443. doi: 10.1111/j.1467-8489.2004.00258.x

[22] Chomitz KM, Alger K, Thomas TS, Orlando H, Nova PV. Opportunity costs of conservation in a biodiversity hotspot: the case of southern Bahia. Environ Dev Econ. 2005;10:293–312. doi: 10.1017/s1355770x05002081 | issn 1355-770x WOS:000229898800003.

[23] Adams VM, Pressey RL, Naidoo R. Opportunity costs: who really pays for conservation. Biol Conserv. 2010;143:439–448.

[24] Naidoo R, Adamowicz WL. Modeling opportunity costs of conservation in transitional landscapes. Conserv Biol. 2006;20(2):490–500. doi: 10.1111/j.1523-1739.2006.00304.x 16903110

[25] Naidoo R, Iwamura T. Global-scale mapping of economic benefits from agricultural lands: Implications for conservation priorities. Biol Conserv. 2007;140(1–2):40–49. ISI:000250903000005.

[26] Adams VM, Mills M, Jupiter SD, Pressey RL. Improving social acceptability of marine protected area networks: a method for estimating opportunity costs to multiple gear types in both fished and currently unfished areas. Biol Conserv. 2011;144:350–361.

[27] Ban NC, Klein CJ. Spatial socioeconomic data as a cost in systematic marine conservation planning. Conserv Lett. 2009;2(5):206–215.

[28] Teixeira JB, Moura RL, Mills M, Klein C, Brown CJ, Adams VM, et al. A novel habitat-based approach to predict impacts of marine protected areas on fishers. Conserv Biol. 2018;32:1096–1106. doi: 10.1111/cobi.12974 28646574

[29] Iacona GD, Sutherland WJ, Mappin B, Adams V, Armsworth PR, Coleshaw T, et al. Standardized reporting of the costs of management interventions for biodiversity conservation. Conserv Biol. 2018;32:979–988. doi: 10.1111/cobi.13195 30039609

[30] Craigie ID, Pressey RL. Fine-grained data and models of protected-area management costs reveal cryptic effects of budget shortfalls. Biol Conserv. 2022;272:109589. doi: 10.1016/j.biocon.2022.109589

[31] Armsworth PR, Cantú-Salazar L, Parnell M, Davies ZG, Stoneman R. Management costs for small protected areas and economies of scale in habitat conservation. Biol Conserv. 2011;144(1):423–429.

[32] Armsworth PR, Jackson HB, Cho S-H, Clark M, Fargione JE, Iacona GD, et al. Factoring economic costs into conservation planning may not improve agreement over priorities for protection. Nat Commun. 2017;8(1):2253. doi: 10.1038/s41467-017-02399-y 29269829 PMC5740120

[33] Sacre E, Pressey RL, Bode M. Costs are not necessarily correlated with threats in conservation landscapes. Conserv Lett. 2019;12(5):e12663.

[34] Sacre E, Weeks R, Bode M, Pressey RL. The relative conservation impact of strategies that prioritize biodiversity representation, threats, and protection costs. Conserv Sci Pract. 2020;2(8):e221.

[35] Cheok J, Pressey RL, Weeks R, Andréfouët S, Moloney J. Sympathy for the Devil: Detailing the Effects of Planning-Unit Size, Thematic Resolution of Reef Classes, and Socioeconomic Costs on Spatial Priorities for Marine Conservation. PLoS ONE. 2016;11(11):e0164869. doi: 10.1371/journal.pone.0164869 27829042 PMC5102401

[36] Newburn D, Reed S, Berck P, Merenlender A. Economics and land-use change in prioritizing private land conservation. Conserv Biol. 2005;19(5):1411–1420. doi: 10.1111/j.1523-1739.2005.00199.x | ISSN 0888-8892 WOS:000232137900010.

[37] Arponen A, Cabeza M, Eklund J, Kujala H, LehtomÄKi J. Costs of Integrating Economics and Conservation Planning. Conserv Biol. 2010;24(5):1198–1204. doi: 10.1111/j.1523-1739.2010.01539.x 20575989

[38] Conservation Measures Partnership. Open Standards for the Practice of Conservation Version4.0. Available from: https://conservationstandards.org/. 2020.

[39] Game ET, Kareiva P, Possingham HP. Six common mistakes in conservation priority setting. Conserv Biol. 2013;27(3):480–485. doi: 10.1111/cobi.12051 23565990 PMC3732384

[40] Wilson KA, Carwardine J, Possingham HP. Setting conservation priorities. Year in Ecology and Conservation Biology. 2009;1162:237–264. doi: 10.1111/j.1749-6632.2009.04149.x ISI:000266235600010. 19432651

[41] Evans MC, Tulloch AIT, Law EA, Raiter KG, Possingham HP, Wilson KA. Clear consideration of costs, condition and conservation benefits yields better planning outcomes. Biol Conserv. 2015;191:716–727. doi: 10.1016/j.biocon.2015.08.023

[42] Margoluis R, Stem C, Swaminathan V, Brown M, Johnson A, Placci G, et al. Results chains: a tool for conservation action design, management, and evaluation. Ecol Soc. 2013;18(3). doi: 10.5751/es-05610-180322

[43] Adams VM, Mills M, Weeks R, Segan DB, Pressey RL, Gurney GG, et al. Implementation strategies for systematic conservation planning. Ambio. 2019;48(2):139–152. doi: 10.1007/s13280-018-1067-2 29949079 PMC6346603

[44] Reed MS. Stakeholder participation for environmental management: A literature review. Biol Conserv. 2008;141(10):2417–2431. doi: 10.1016/j.biocon.2008.07.014

[45] Reed MS, Graves A, Dandy N, Posthumus H, Hubacek K, Morris J, et al. Who’s in and why? A typology of stakeholder analysis methods for natural resource management. J Environ Manage. 2009;90(5):1933–1949. doi: 10.1016/j.jenvman.2009.01.001 19231064

[46] Adams WM, Aveling R, Brockington D, Dickson B, Elliott J, Hutton J, et al. Biodiversity conservation and the eradication of poverty. Science. 2004;306(5699):1146–1149. doi: 10.1126/science.1097920 15539593

[47] Sutton NJ, Cho S, Armsworth PR. A reliance on agricultural land values in conservation planning alters the spatial distribution of priorities and overestimates the acquisition costs of protected areas. Biol Conserv. 2016;194:2–10. doi: 10.1016/j.biocon.2015.11.021

[48] Balmford A, Gravestock P, Hockley N, McClean CJ, Roberts CM. The worldwide costs of marine protected areas. Proc Natl Acad Sci U S A. 2004;101(26):9694–9697. doi: 10.1073/pnas.0403239101 WOS:000222405600033. 15205483 PMC470737

[49] Sutton NJ, Armsworth PR. The Grain of Spatially Referenced Economic Cost and Biodiversity Benefit Data and the Effectiveness of a Cost Targeting Strategy. Conserv Biol. 2014:28 1451–61. doi: 10.1111/cobi.12405 25381868

[50] McCreless E, Visconti P, Carwardine J, Wilcox C, Smith RJ. Cheap and Nasty? The Potential Perils of Using Management Costs to Identify Global Conservation Priorities. PLoS ONE. 2013;8(11):e80893. doi: 10.1371/journal.pone.0080893 24260502 PMC3829910

[51] Yong C, Ward M, Watson JEM, Reside AE, van Leeuwen S, Legge S, et al. The costs of managing key threats to Australia’s biodiversity. J Appl Ecol. 2023;60(5):898–910. doi: 10.1111/1365-2664.14377

[52] Anna Bligh Premier of Queensland. Witches Falls’ 100th birthday; national park area increase commitment [Transcript Speech]. (accessed 2010 Feb) 2008 [updated 2008 Mar 28]. Available from: http://www.thepremier.qld.gov.au/library/word/newsroom/video/Witches Falls transcript.doc.

[53] Taylor MFJ, Adams VM, Segan DB, Pressey RL. 20 million hectares by 2020: Protected areas, green infrastructure and green jobs for Queensland. Sydney: 2009.

[54] Cook CN, Pullin AS, Sutherland WJ, Stewart GB, Carrasco LR. Considering cost alongside the effectiveness of management in evidence-based conservation: A systematic reporting protocol. Biol Conserv. 2017;209:508–516. doi: 10.1016/j.biocon.2017.03.022

[55] Armsworth PR, Daily GC, Kaireva P, Sanchirico JN. Land market feedbacks can undermine biodiversity conservation. Proc Natl Acad Sci U S A. 2006;103(14):5403–5408. doi: 10.1073/pnas.0505278103 16554375 PMC1459367

[56] Hanley N, Czajkowski M. The Role of Stated Preference Valuation Methods in Understanding Choices and Informing Policy. Rev Env Econ Policy. 2019;13(2):248–266. doi: 10.1093/reep/rez005

[57] Murdoch W, Polasky S, Wilson KA, Possingham HP, Kareiva P, Shaw R. Maximizing return on investment in conservation. Biol Conserv. 2007;139:375–88. doi: 10.1016/j.biocon.2007.07.011 | ISSN 0006-3207 WOS:000250330700013.

[58] Yoon HS, Armsworth PR. Timing land protection to exploit favorable market conditions. Biol Conserv. 2022;270:109566. doi: 10.1016/j.biocon.2022.109566

[59] Ban NC, Adams VM, Pressey RL, Hicks J. Promise and problems for estimating management costs of marine protected areas. Conserv Lett. 2011;4:241–252.

[60] Adams VM, Pressey RL, Stoeckl N. Estimating land and conservation management costs: the first step in designing a stewardship program for the Northern Territory. Biol Conserv. 2012;148:44–53.

[61] Wenger AS, Adams VM, Iacona GD, Lohr C, Pressey RL, Morris K, et al. Estimating realistic costs for strategic management planning of invasive species eradications on islands. Biol Invasions. 2017. doi: 10.1007/s10530-017-1627-6

[62] Adams VM, Douglas MM, Jackson S, Scheepers K, Kool J, Setterfield SA. Conserving biodiversity and Indigenous bush tucker: Practical application of the strategic foresight framework to invasive alien species management planning. Conserv Lett. 2018;2018:e12441. doi: 10.1111/conl.12441

[63] McMaster D, Adams VM, Setterfield SA, McIntyre D, Douglas MM, editors. Para grass management and costing trial within Kakadu National Park. 19th Australasian Weeds Conference; 2014; Hobart, Tasmania: Weed Society of Tasmania Inc.

[64] Rossiter-Rachor NA, Adams VM, Canham CA, Dixon DJ, Cameron TN, Setterfield SA. The cost of not acting: Delaying invasive grass management increases costs and threatens assets in a national park, northern Australia. J Environ Manage. 2023;333:116785. doi: 10.1016/j.jenvman.2022.116785 36758396

[65] Cacho OJ, Wise RM, Hester SM, Sinden JA. Bioeconomic modeling for control of weeds in natural environments. Ecol Econ. 2008;65(3):559–568. doi: 10.1016/j.ecolecon.2007.08.006

[66] Panetta FD, Cacho OJ, Hester SM, Sims-Chilton NM. Estimating the duration and cost of weed eradication programmes. In: Veitch CR, Clout MN, Towns DR, editors. Island invasives: eradication and management. Gland, Switzerland: IUCN; 2011. p. 472–6.

[67] Cacho OJ, Spring D, Hester S, Mac NR. Allocating surveillance effort in the management of invasive species: A spatially-explicit model. Environ Model Software. 2010;25(4):444–454. doi: 10.1016/j.envsoft.2009.10.014

[68] Adams VM, Petty AM, Douglas MM, Buckley YM, Ferdinands KB, Okazaki T, et al. Distribution, demography and dispersal model of spatial spread of invasive plant populations with limited data. Methods Ecol Evol. 2015;6(7):782–794.

[69] Adams VM, Setterfield SA. Approaches to strategic risk analysis and management of invasive plants: lessons learned from managing gamba grass in northern Australia. Pac Conserv Biol. 2016;22:189–200. doi: 10.1071/PC15041

[70] Adams VM, Setterfield SA. Optimal dynamic control of invasions: applying a systematic conservation approach. Ecol Appl. 2015;25(4):1131–1141. doi: 10.1890/14-1062.1 26465047

[71] Jindal R, Kerr JM, Ferraro PJ, Swallow BM. Social dimensions of procurement auctions for environmental service contracts: Evaluating tradeoffs between cost-effectiveness and participation by the poor in rural Tanzania. Land Use Policy. 2013;31(0):71–80. doi: 10.1016/j.landusepol.2011.11.008

[72] Munoz-Pina C, Guevara A, Torres JM, Brana J. Paying for the hydrological services of Mexico’s forests: Analysis, negotiations and results. Ecol Econ. 2008;65(4):725–36. doi: 10.1016/j.ecolecon.2007.07.031 WOS:000256151100006.

[73] Ferraro PJ. Asymmetric information and contract design for payments for environmental services. Ecol Econ. 2008;65(4):810–821. doi: 10.1016/j.ecolecon.2007.07.029 WOS:000256151100013.

[74] Babcock BA, Lakshminarayan PG, Wu J, Zilberman D. The economics of a public fund for environmental amenities: a study of CRP contracts. (Conservation Reserve Program). Am J Agric Econ. 1996;v78(n4):p961(11).

[75] Hajkowicz S, Higgins A, Williams K, Faith DP, Burton M. Optimisation and the selection of conservation contracts. Aust J Agric Resour Econ. 2007;51(1):39–56. doi: 10.1111/j.1467-8489.2007.00345.x ISI:000243533100003.

[76] Stoneham G, Chaudhri V, Ha A, Strappazzon L. Auctions for conservation contracts: an empirical examination of Victoria’s BushTender trial. Aust J Agric Resour Econ. 2003;47(4):477–500.

[77] Jackson HB, Kroetz K, Sanchirico JN, Thompson A, Armsworth PR. Protected area, easement, and rental contract data reveal five communities of land protection in the United States. Ecol Appl. 2021;31(5):e02322. doi: 10.1002/eap.2322 33655588

[78] Du Toit DR, Biggs H, Pollard S. The Potential Role of Mental Model Methodologies in Multistakeholder Negotiations: Integrated Water Resources Management in South Africa. Ecol Soc. 2011;16(3). doi: 10.5751/es-04237-160321

[79] Pressey R. Algorithms, politics and timber: an example of the role of science in a public, political negotiation process over new conservation areas in production forests. In: Wills R, Hobbs R, editors. Ecology for everyone: communicating ecology to scientists, the public and the politicians. Sydney: Surrey Beatty and Sons; 1998. p. 73–87.

[80] Cinner JE, Wamukota A, Randriamahazo H, Rabearisoa A. Toward institutions for community-based management of inshore marine resources in the Western Indian Ocean. Mar Policy. 2009;33(3):489–496.

[81] Clarke P, Jupiter SD. Law, custom and community-based natural resource management in Kubulau District, Republic of Fiji Islands. Environ Conserv. 2010;37:98–106.

[82] Ban NC, Picard CR, Vincent ACJ. Comparing and integrating community-based and science-based approaches to prioritizing marine areas for protection. Conserv Biol. 2009;23(4):899–910. doi: 10.1111/j.1523-1739.2009.01185.x ISI:000268028800021. 19627319

[83] Weeks R, Jupiter SD. Adaptive Comanagement of a Marine Protected Area Network in Fiji. Conserv Biol. 2013;27(6):1234–1244. doi: 10.1111/cobi.12153 24112643 PMC4232917

